# Identification of long noncoding RNAs involved in muscle differentiation

**DOI:** 10.1371/journal.pone.0193898

**Published:** 2018-03-02

**Authors:** Yeong-Hwan Lim, Duk-Hwa Kwon, Jaetaek Kim, Woo Jin Park, Hyun Kook, Young-Kook Kim

**Affiliations:** 1 Basic Research Laboratory for Cardiac Remodeling Research Laboratory, Chonnam National University Medical School, Jeollanam-do, Republic of Korea; 2 Department of Biochemistry, Chonnam National University Medical School, Jeollanam-do, Republic of Korea; 3 Department of Biomedical Sciences, Center for Creative Biomedical Scientists at Chonnam National University, Jeollanam-do, Republic of Korea; 4 Department of Pharmacology, Chonnam National University Medical School, Jeollanam-do, Republic of Korea; 5 Division of Endocrinology and Metabolism, Department of Internal Medicine, College of Medicine, Chung-Ang University, Seoul, Republic of Korea; 6 College of Life Sciences, Gwangju Institute of Science and Technology (GIST), Gwangju, Republic of Korea; University of Nevada School of Medicine, UNITED STATES

## Abstract

Long noncoding RNAs (lncRNAs) are a large class of regulatory RNAs with diverse roles in cellular processes. Thousands of lncRNAs have been discovered; however, their roles in the regulation of muscle differentiation are unclear because no comprehensive analysis of lncRNAs during this process has been performed. In the present study, by combining diverse RNA sequencing datasets obtained from public database, we discovered lncRNAs that could behave as regulators in the differentiation of smooth or skeletal muscle cells. These analyses confirmed the roles of previously reported lncRNAs in this process. Moreover, we discovered dozens of novel lncRNAs whose expression patterns suggested their possible involvement in the phenotypic switch of vascular smooth muscle cells. The comparison of lncRNA expression change suggested that many lncRNAs have common roles during the differentiation of smooth and skeletal muscles, while some lncRNAs may have opposite roles in this process. The expression change of lncRNAs was highly correlated with that of their neighboring genes, suggesting that they may function as cis-acting lncRNAs. Furthermore, within the lncRNA sequences, there were binding sites for miRNAs with expression levels inversely correlated with the expression of corresponding lncRNAs during differentiation, suggesting a possible role of these lncRNAs as competing endogenous RNAs. The lncRNAs identified in this study will be a useful resource for future studies of gene regulation during muscle differentiation.

## Introduction

The major type of smooth muscle cells, vascular smooth muscle cells (VSMCs), are capable of converting between synthetic and contractile phenotypes [1, 2]. In the normal state, VSMCs exist as the differentiated and contractile type. However, in response to injury, VSMCs become de-differentiated into a more proliferative and synthetic phenotype. Diverse factors and complex regulatory networks govern the phenotypic change of VSMCs [3]. Disturbances in the proliferation or differentiation of VSMCs can lead to various vascular diseases, such as atherosclerosis, restenosis, and hypertension, and intensive research is underway to identify the diverse factors involved in this process [4].

In contrast to the reversible change in phenotype of smooth muscle cells, terminally differentiated skeletal muscle, like many other cell types, cannot be dedifferentiated. Upon external signals such as muscle injury, skeletal muscle is regenerated from satellite cells, which are main myogenic progenitor cells. The satellite cells are divided asymmetrically, and expanded into myoblasts, which are further differentiated into myocytes. This differentiation process is influenced by diverse factors, and for the treatment of diseases such as skeletal muscle degeneration, the elucidation of signaling networks involved in this process is critical [5, 6].

Many studies have shown that various types of noncoding RNAs have regulatory roles in most cellular processes. MicroRNAs (miRNAs), a class of small noncoding RNAs, have been studied extensively for a long time [7]. The regulatory roles of dozens of miRNAs have been reported for diverse aspects of smooth muscle cells [8]. Long noncoding RNAs (lncRNAs) are another group of regulatory RNAs that are longer than 200 nucleotides but lack protein-coding potential [9]. Compared with miRNAs, only a few studies have identified the roles of lncRNAs for smooth muscle, including the regulation of proliferation or apoptosis. One of the first identified lncRNAs was the smooth muscle and endothelial cell-enriched migration/differentiation-associated lncRNA (*SENCR*) [10]. Inhibition of SENCR increased the migration of smooth muscle cells. Another lncRNA essential for the proliferation of VSMCs is smooth muscle-induced lncRNA enhances replication (*SMILR*) [11]. SMILR regulates its neighboring protein-coding gene, hyaluronan synthase 2 (*HAS2*), an enzyme that synthesizes hyaluronic acid. The function of only a small number of lncRNAs has been elucidated in the phenotypic change of VSMCs; therefore, it is hypothesized that additional lncRNAs involved in this process await discovery. To date, various reagents have been used to model the differentiation process of VSMCs, such as platelet-derived growth factor (PDGF), myocardin (MYOCD), transforming growth factor beta (TGFβ), activin A, retinoids, and angiotensin II. The transcriptome responds quite differentially to each of these factors; therefore, to identify common lncRNAs, diverse experimental models should be combined.

In the present study, we aimed to comprehensively discover lncRNAs involved in muscle differentiation by combining diverse RNA sequencing (RNA-seq) datasets. By integrating the data from VSMCs where different extracellular cues were used for differentiation, we identified several lncRNAs with potential as regulators in VSMCs differentiation. We analyzed the correlation between the expression of lncRNAs and that of their neighboring protein-coding genes, and compared the expression change of lncRNAs between smooth and skeletal muscle cells.

## Materials and methods

### Analysis of RNA-seq data

We obtained RNA-seq data from the Gene Expression Omnibus (GEO) database [12]. These data included RNA-seq data from platelet-derived growth factor (PDGF)-treated venous smooth muscle cells (GSE69637), myocardin (MYOCD)-overexpressed human coronary artery smooth muscle (HCASM) cells (GSE77120), transforming growth factor beta (TGFβ)-treated HCASM cells (GSE85910), and human myoblasts differentiated with low serum (GSE70389). The Trimmomatic algorithm was used to remove adaptor sequences and filter the reads with low quality (option: ILLUMINACLIP:TruSeq3-PE-2.fa:2:30:10 LEADING:3 TRAILING:3 SLIDINGWINDOW:4:15) [13]. Reads were aligned to the human genome (hg19) using STAR with default options [14]. SAMtools was used to remove reads with low mapping quality (option: -q 10) [15]. To calculate the FPKM (fragments per kilobase of exon model per million mapped fragments), Cuffnorm was used together with the GENCODE annotation for lncRNAs (GRCh37, release 26; http://www.gencodegenes.org/) [16], and the annotation of UCSC genes for protein-coding genes (hg19; http://genome.ucsc.edu/) [17]. The GENCODE annotation contains information for 15,901 lncRNAs. We removed the lncRNAs with an average FPKM value in each dataset lower than 5. This filtering resulted in 2,361 lncRNAs remaining for further analysis.

### Selection of candidate lncRNAs involved in muscle differentiation

To select the lncRNAs with differential expression during differentiation of VSMCs, we selected only those lncRNAs with a log2 value of expression changes greater than 0.5 or lower than -0.5 between before and after treatment with external factors. To select the lncRNAs that increased in the synthetic phenotype, those lncRNAs with increased expression in the PDGF-treated sample, but with decreased expression in the MYOCD- and TGFβ-treated samples, were chosen. In the case of the lncRNAs that increased in the contractile phenotype, we chose those lncRNAs with decreased expression in the PDGF-treated sample, but with increased expression in the MYOCD- and TGFβ-treated samples. We removed the lncRNAs from the list if the FPKM values of ≥ 50% of the samples belonging to the denominator were zero. To select statistically significant lncRNAs, a two-tailed T-test was applied to each dataset, and labeled as ‘significant’ if the *p* values were less than 0.1 in more than two datasets and less than 0.3 in all datasets.

To select lncRNAs that are differentially expressed during skeletal muscle differentiation (GSE70389), we used the same criteria that were used for lncRNA selection in VSMCs, except that the *p* value criteria in the T-test as 0.2.

### *In silico* promoter analysis

To predict transcription factors that might regulate the expression of lncRNAs, we used the same approach as that detailed in our previous report for the analysis of miRNA promoters [18]. We used the data released by the ENCODE project, where chromatin immunoprecipitation followed by sequencing (ChIP-seq) is performed for 161 transcription factors [19]. We selected the genomic region spanning from -2,000 to +500 nucleotides against the 5′ end of the lncRNA as the promoter of the lncRNA. For the lncRNAs with incomplete annotation in GENCODE, we modified the annotation by comparison with the information from RefSeq. After collecting the genomic coordinates of the lncRNA promoters, we searched for ChIP-seq signals of transcription factors within this region.

### Measurement of RNAs during the differentiation of HCASM

To measure the expression level of lncRNAs and their neighboring protein-coding genes during differentiation of smooth muscle cells, we cultured HCASM cells (Gibco) according to the manufacturer’s protocol. These cells were maintained in Medium 231 (Gibco) supplemented with Smooth Muscle Growth Supplement (Gibco), and harvested for the sample of synthetic phenotype. To obtain the sample of contractile or differentiated phenotype, HCASM cells stabilized in the growth media were cultured in Medium 231 supplemented with Smooth Muscle Differentiation Supplement (Gibco) for three days, and then collected. We used TRIzol Reagent (Invitrogen) to extract total RNA, performed reverse transcription using RevertAid Reverse Transcriptase (Thermo Scientific) with Random Hexamers (Invitrogen). Quantitative real time polymerase chain reaction (PCR) was performed using SYBR Green PCR Master Mix (Applied biosystems) in Rotor-Gene Q (Qiagen). The primer sequences for PCR were included in the supplementary table. The differentiation of these cells was confirmed by measuring the level of Calponin 1 (CNN1) and Smooth muscle protein 22-alpha (SM22α). For normalization, the expression level of Glyceraldehyde 3-phosphate dehydrogenase (GADPH) was used.

### Analysis of lncRNAs as miRNA regulators

In the track data hubs of UCSC Genome Browser [17], we utilized miRcode, which is a prediction algorithm of human miRNA targets based on GENCODE annotation including lncRNAs [20]. We intersected the miRcode track with lncRNA track using Table browser, to collect the list of lncRNA-targeting miRNAs. To select more reliable miRNAs, we obtained miRNA microarray data for human VSMCs undergoing phenotypic switching in response to serum withdrawal (GSE19544) [21]. Among the miRNAs predicted to target lncRNAs, we only selected the miRNAs with expression inversely correlated with lncRNAs during VSMCs differentiation.

## Results and Discussion

### Identification of lncRNAs involved in phenotypic change of VSMCs

**Fig 1:**
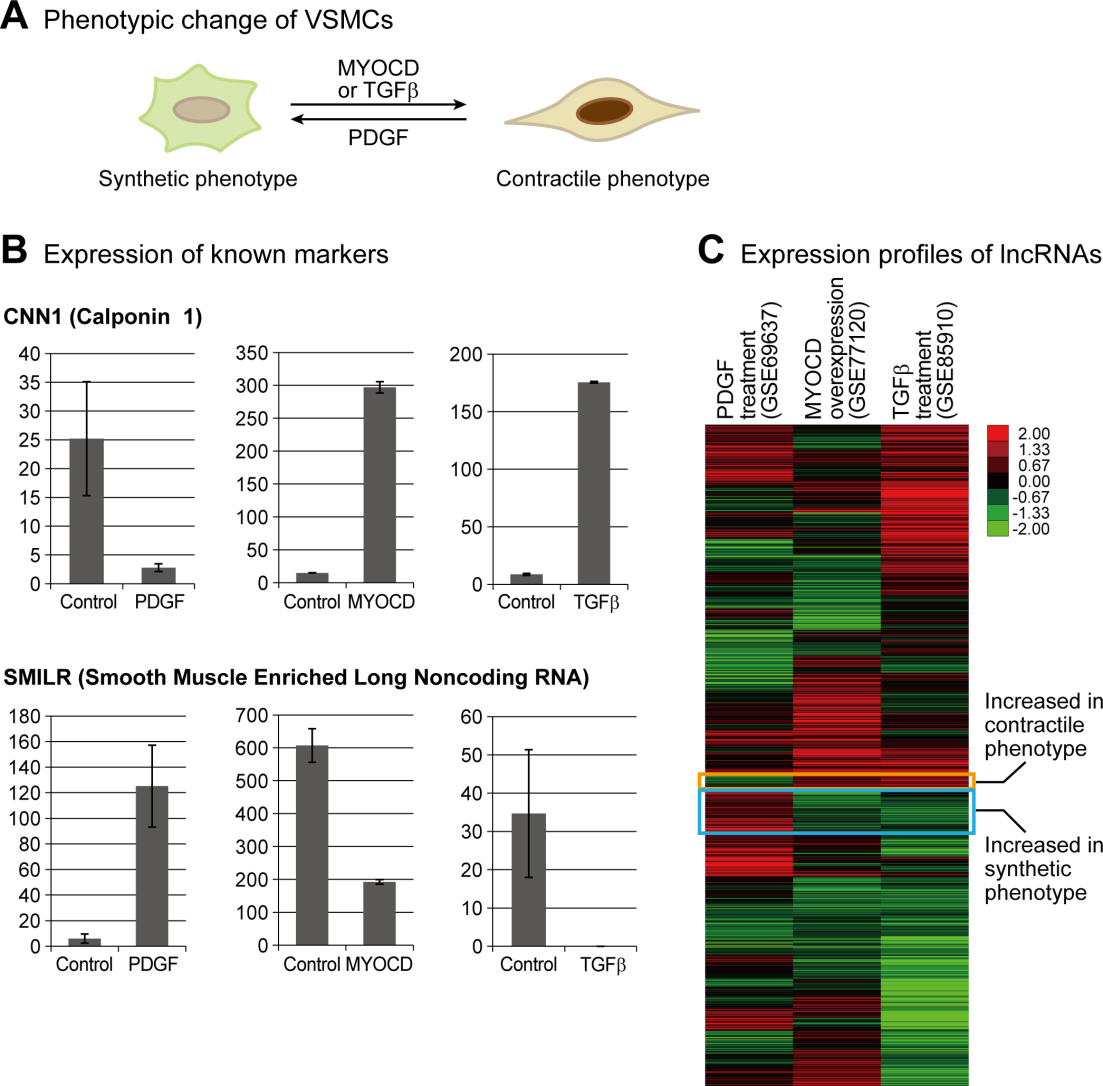
Identification of long noncoding RNAs (lncRNAs) involved in phenotypic change of vascular smooth muscle cells (VSMCs). (A) The VSMCs with the synthetic phenotype can be differentiated into less the proliferative and contractile phenotypic by the overexpression of myocardin (MYOCD) or by treatment with transforming growth factor beta (TGFβ). The cells with the contractile phenotype can be converted into the more proliferative and synthetic phenotype by treatment with platelet-derived growth factor (PDGF). (B) Expression level of representative genes previously known to be involved in phenotypic change of VSMCs. The RNA-seq data of PDGF-treated venous smooth muscle cells, MYOCD-overexpressing human coronary artery smooth muscle (HCASM) cells, and TGFβ-treated HCASM cells were obtained from the Gene Expression Omnibus (GEO) (see Methods). The fragments per kilobase of exon model per million mapped fragments (FPKM) values of a protein-coding gene (*CNN1*) and a long noncoding RNA (*SMILR*) are depicted in the y-axis. Error bars indicate standard errors from four samples for the PDGF set, or deviation from two samples for the MYOCD and TGFβ sets. (C) Expression profiles of lncRNAs during phenotypic change of VSMCs were analyzed. The calculation of lncRNAs’ expression changes is described in Method section. The gene clusters with prominent changes between the synthetic and contractile phenotypes are indicated with boxes. Detailed expression changes of genes from those clusters are depicted in S1 and S2 Figs.

VSMCs can be switched between synthetic and contractile phenotypes in response to diverse extracellular cues [1, 2]. To discover lncRNAs involved in the regulation of this process, we obtained RNA-seq data from the GEO database [12]. First, RNA-seq data was obtained from VSMCs treated with PDGF and their untreated controls (GSE69637) [11]. PDGF induces the synthetic phenotype of VSMCs (Fig 1A) [1]. Data from HCASMs overexpressing myocardin (MYOCD) (GSE77120) and those cells treated with TGFβ (GSE85910) were obtained [22]. Both MYOCD and TGFβ were reported to induce a contractile phenotype (Fig 1A) [1, 2]. We integrated the data from artery and venous smooth muscles; therefore, it was possible to identify common lncRNAs involved in the differentiation of both types of smooth muscles. We collected these datasets and analyzed the expression levels of lncRNAs and protein-coding genes. We confirmed the phenotypic change of VSMCs from the treatment of each factor by calculating the expression level of CNN1 and smooth muscle-induced lncRNA enhances replication (SMILR) (Fig 1B). CNN1 was previously reported to increase, while SMILR is known to decrease, in the contractile phenotype of VSMCs [1, 11]. Next, we measured the expression of whole lncRNAs annotated in the recent release of the GENCODE database (release 26), and compared their expression changes during the phenotypic change of VSMCs (S1 Table). We found that several lncRNAs showed consistent differential expression between the synthetic and contractile phenotype of VSMCs in the three datasets (Fig 1C and S1 and S2 Figs). However, when we calculated the correlation for whole lncRNAs, there was almost no correlation among the three datasets (the correlation coefficients ranged from 0.02 to 0.04). Thus, most lncRNAs are not directly related with the phenotypic change of VSMCs. Otherwise, they might have a specific role during phenotypic change induced by different contexts. This result suggests that it is crucial to integrate the data from diverse datasets to identify common factors involved in a physiological process such as VSMCs differentiation.

**Fig 2:**
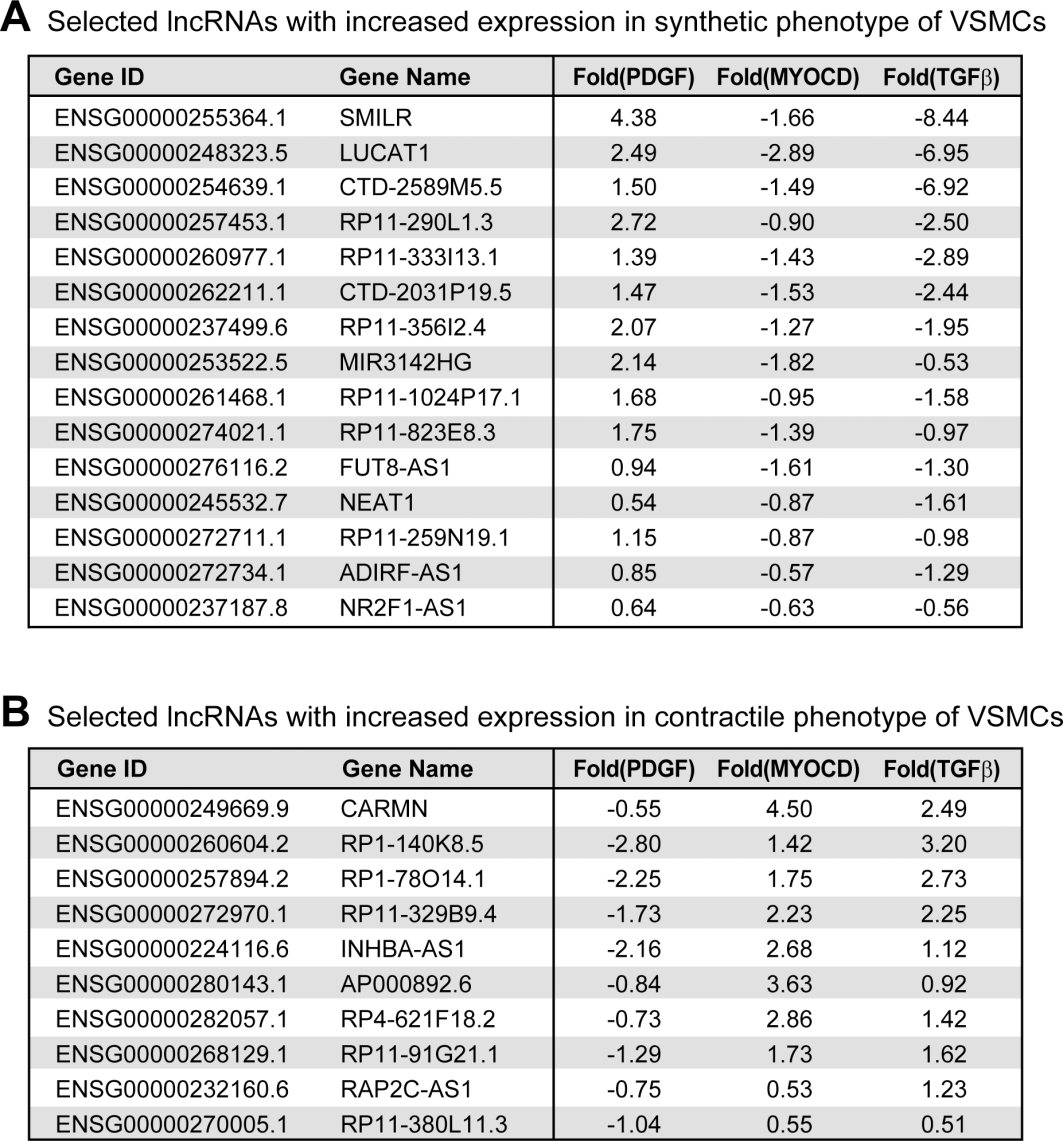
Selected long noncoding RNAs (lncRNAs) with differential expression between synthetic and contractile phenotypes of vascular smooth muscle cells (VSMCs). Based on the expression change of lncRNAs between the synthetic and contractile phenotypes from three different datasets, we selected highly reliable lncRNAs that could be involved in the regulation of phenotypic switch of VSMCs. The lncRNAs with increased expression in synthetic (A) and contractile (B) phenotypes are listed in descending order of their changes in expression, respectively. For each lncRNA, their annotated gene name and log_2_(fold change) in each dataset are shown. The fragments per kilobase of exon model per million mapped fragments (FPKM) values of lncRNAs in each sample are summarized in S1 Table.

To identify those lncRNAs that are likely to play a role in phenotypic change of VSMCs, we statistically tested for the selected lncRNAs shown in S1 Table, and listed only those lncRNAs with significantly changed expression among the three datasets (Fig 2). This list included 15 lncRNAs increased in synthetic phenotype and 10 lncRNAs increased in contractile phenotype. The most highly changed lncRNAs in each direction of phenotypic conversion were SMILR and cardiac mesoderm enhancer-associated non-coding RNA (CARMN), respectively. SMILR was reported to be a regulator of smooth muscle cells differentiation, while the role of CARMN was identified from cardiac cells [11, 23]. As a host gene of miR-143 and miR-145, CARMN is required to maintain the differentiated state of cardiomyocytes. Thus, it may be expected that CARMN is involved in the differentiation of both smooth and cardiac muscles. The second most increased lncRNA in the synthetic phenotype of VSMCs, lung cancer associated transcript 1 (LUCAT1), was identified as an lncRNA that increased in human pulmonary artery smooth muscle cells exposed to hypoxia, although its role in this process was not studied [24]. In addition, nuclear enriched abundant transcript 1 (NEAT1), one of the increased lncRNAs in the synthetic phenotype, has been implicated in the differentiation of skeletal muscle, as knockdown of NEAT1 increased the expression of marker genes of muscle differentiation in C2C12 cells [25]. Among the other lncRNAs in the list, *MIR3142HG* is the host gene of miR-3142 and miR-146a. miR-146a regulates the differentiation of VSMCs by targeting nuclear factor-κBp65 (NF-κBp65) and the proliferative cell nuclear antigen (PCNA) [26]. Thus, it is possible that the expression change in the *MIR3142HG* gene results in the change in miR-146a level, which regulates the physiology of VSMCs, although we could not exclude a miRNA-independent role of this lncRNA. For the other lncRNAs, their roles in muscle physiology have not been reported yet. Thus, the lncRNAs identified in this analysis represent good candidates for future studies to elucidate their roles during muscle differentiation.

To identify any transcription factors with enriched binding into the promoter regions of differentially expressed lncRNAs, we used the data released by the ENCODE project, where ChIP-seq was performed for 161 major transcription factors [19]. Among the transcription factors with ChIP-seq signals, SIN3A, SMC3, and RAD21 showed significant enrichment in the promoters of lncRNAs increased in the synthetic phenotype (S2 Table). A previous report showed that the transcriptional repressor, SIN3A, is required for the development of skeletal muscle [27], although its role in smooth muscle is unknown. Further studies are required to test whether the transcriptional regulation of lncRNAs by SIN3A, and also by SMC3 and RAD21, is involved in the phenotypic switch of VSMCs.

### Comparison of lncRNAs between smooth and skeletal muscles

**Fig 3:**
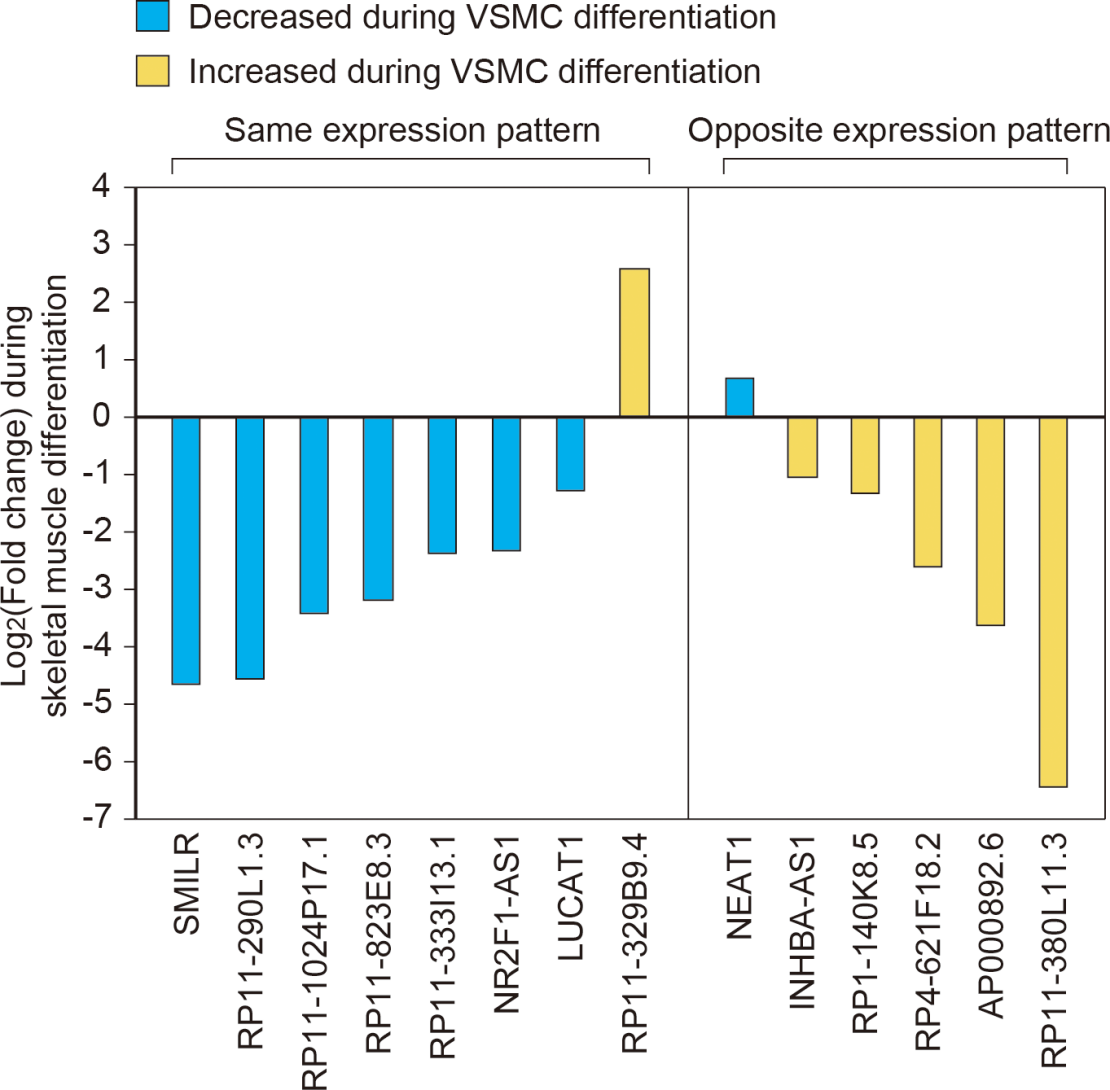
Comparison of long noncoding RNA (lncRNAs) expression between smooth and skeletal muscles. For those lncRNAs with reliable expression levels in both the smooth and skeletal muscle datasets, and with significant expression change during differentiation of vascular smooth muscle cells (VSMCs), their log_2_(fold change) values were compared between smooth and skeletal muscle datasets. Those lncRNAs that show the same direction of expression change during differentiation in both datasets are depicted on the left. Those lncRNAs that increased during the differentiation of smooth muscle but decreased during the differentiation in skeletal muscle, or vice versa, are shown on the right.

Although smooth and skeletal muscles have different morphologies and exert dissimilar roles in the body, they share several factors during their differentiation, such as serum response factor (SRF) [28]. To identify the lncRNAs that show similar changes during the differentiation of these two muscle types, we obtained the RNA-seq data from GEO (GSE70389), which compared the transcriptome between myoblasts and myotubes during skeletal muscle differentiation [29]. We found that among 14 lncRNAs with meaningful expression in both smooth and skeletal muscles, eight lncRNAs showed a similar pattern of expression change in both muscle types (Fig 3). For example, one of the lncRNAs with the most drastic expression change during skeletal muscle differentiation, *SMILR*, has a high probability to function in skeletal muscle, although its role was only reported in smooth muscle [11]. In contrast, six lncRNAs showed opposite direction of expression change between the differentiation of smooth and skeletal muscles (Fig 3). Thus, they may exert a different effect during the differentiation of these two muscle types.

### Confirmation of lncRNAs expression

**Fig 4:**
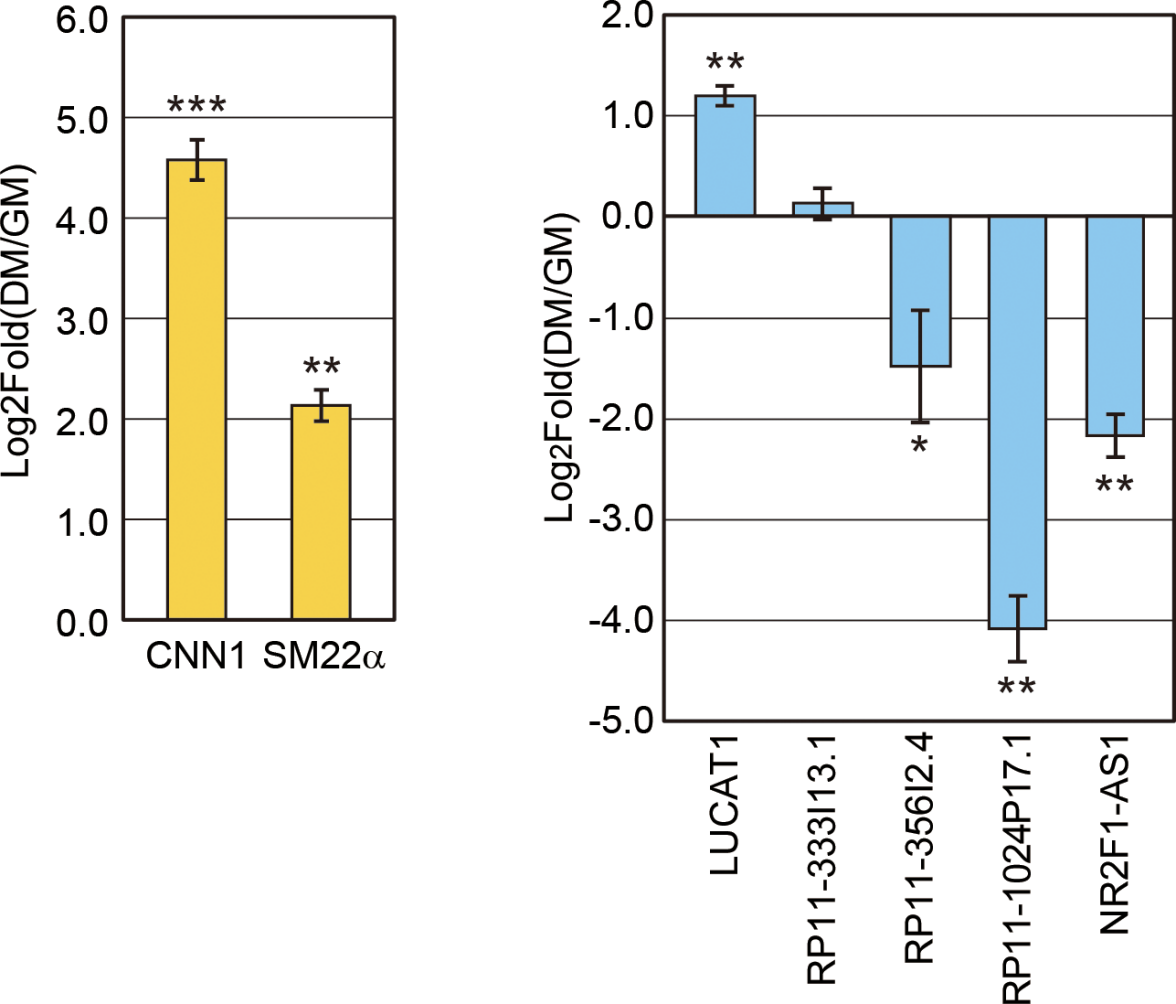
Expression confirmation of lncRNAs during VSMCs differentiation. The expression level of lncRNAs before and after the differentiation of VSMCs was measured. The expression of CNN1 and SM22α, which are known to increase in contractile phenotype compared to synthetic phenotype, was used as the marker of VSMCs differentiation. GM indicates growth media while DM means differentiation media. Error bars indicate the standard error from three biological replicates (n = 3). *P* values were calculated from one-sided paired T test (*: *p* < 0.1, **: *p* < 0.01, ***: *p* < 0.001).

To confirm the expression change of lncRNAs selected above, and to test whether the expression change of those lncRNAs is reproducible in another condition of VSMCs differentiation, we cultured HCASM cells with growth media including growth factors, and induced their differentiation with low concentration of serum and without growth factors (see Methods). Using the samples collected before and after differentiation, we confirmed the differentiation of HCASM by measuring the level of differentiation markers including CNN1 and SM22α (Fig 4). We randomly selected five lncRNAs for the measurement, which are decreased after differentiation (Fig 2) and do not fully overlap with the nearest gene. The expression level of three lncRNAs, RP11-356I2.4, RP11-1024P17.1, and NR2F1-AS1, showed the same pattern of expression change also in our differentiation method (Fig 4). Thus, these lncRNAs consistently decreased in contractile or differentiation phenotype of VSMCs among diverse differentiation conditions. Two lncRNAs, LUCAT1 and RP11-333I13.1, did not decrease after differentiation in our method, suggesting that they respond differentially to diverse differentiation signals.

We utilized Coding Potential Assessment Tool (CPAT) and Coding Potential Calculator (CPC to predict possible coding potential for three lncRNAs confirmed above [30, 31]. The result of analysis suggested that these lncRNAs have very low potential to be translated (S3 Table); although we cannot rule out the possibility that short peptide might be translated from the lncRNA sequences.

### Expression correlation of lncRNAs with their neighboring genes

**Fig 5:**
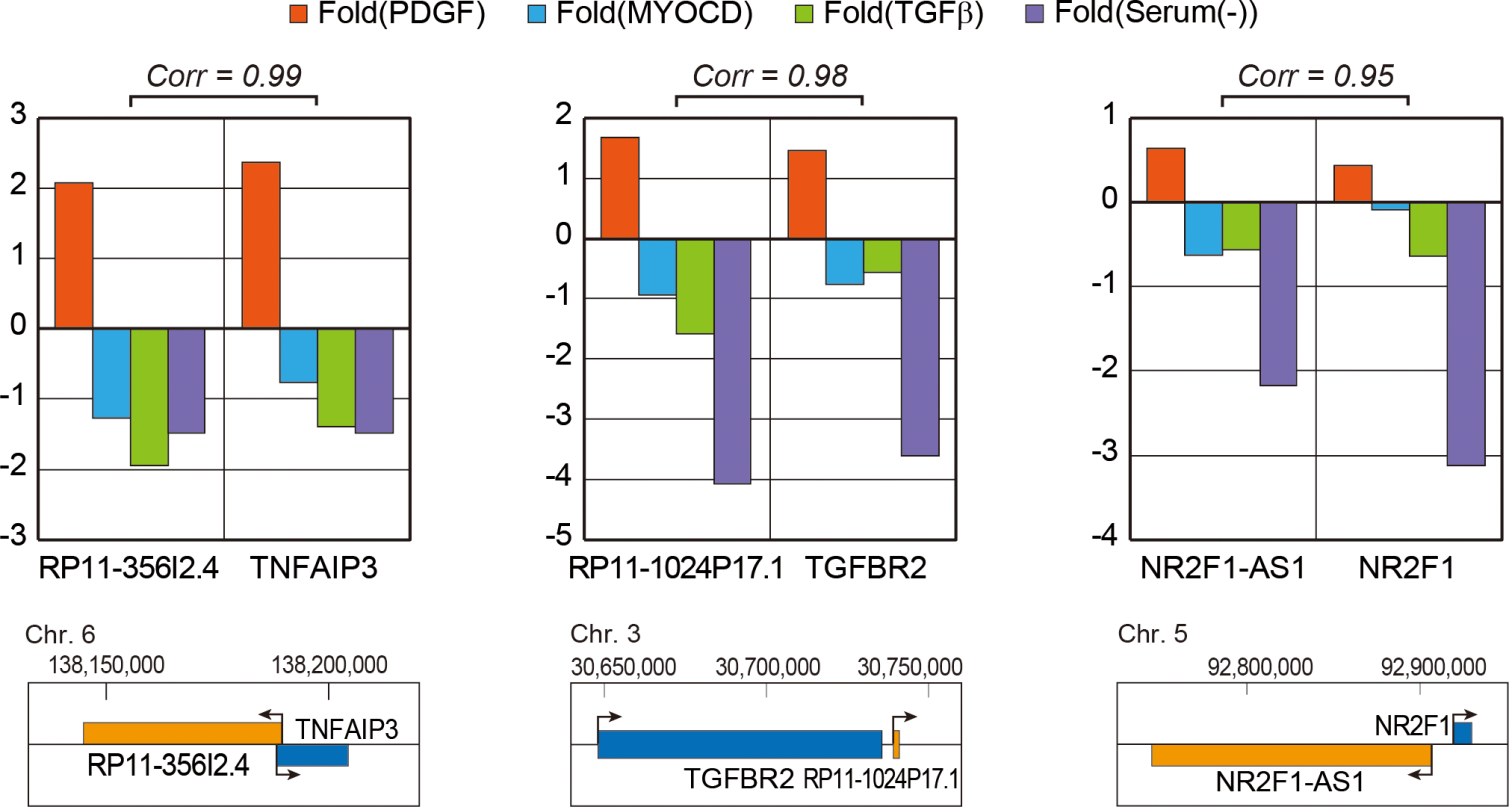
Expression correlation of long noncoding RNAs (lncRNAs) with their nearest genes. The log_2_(fold change) of each lncRNA (left) or its neighboring protein-coding gene (right) is depicted. The fold changes for serum-starved condition were obtained by performing PCR, while other values were calculated using RNA-seq data. The correlation of fold change for each pair of lncRNA and its neighboring gene was calculated and shown above the bar graph. Below each graph, the genomic locations of the lncRNA and its neighboring gene, with their direction of transcription, are indicated. Note that the lncRNA locus is indicated by an orange box, while that of its neighboring protein-coding gene is indicated by a blue box.

Previous studies that elucidated the working mechanisms of lncRNAs suggested that many lncRNAs regulate the expression of their neighboring genes in the genomic context [9]. These ‘cis-acting’ lncRNAs recruit various transcription factors or chromatin remodeling complexes to change the transcription status of nearby genes. Accordingly, the expression levels between those lncRNAs and their neighboring genes are highly correlated. To test whether the lncRNAs identified in the present study behave in a similar way, we checked the expression of the nearest protein-coding gene for each lncRNA. We selected three lncRNAs whose expression change is confirmed in our system (Fig 4). Using the expression data from three RNA-seq datasets of smooth muscle differentiation (Fig 2) and PCR experiments (Fig 4), we calculated the correlation in fold change for lncRNA-neighboring gene pairs (Fig 5). Interestingly, all three pairs showed very high correlation in their expression changes during the differentiation of VSMCs. The data suggests that these lncRNAs may operate by regulating their nearby protein-coding genes. For example, the neighboring gene of lncRNA *RP11-1024P17*.*1*, is *TGFBR2* (encoding TGF beta receptor 2), which has essential roles in vascular development [32, 33]. Thus, the protein-coding genes near the identified lncRNAs tend to be functionally related to the development of VSMCs. Future studies to elucidate the connection between the lncRNA-neighboring gene pairs is essential to understand the working mechanism of these lncRNAs.

### Analysis of the selected lncRNAs as competing endogenous RNAs

**Fig 6:**
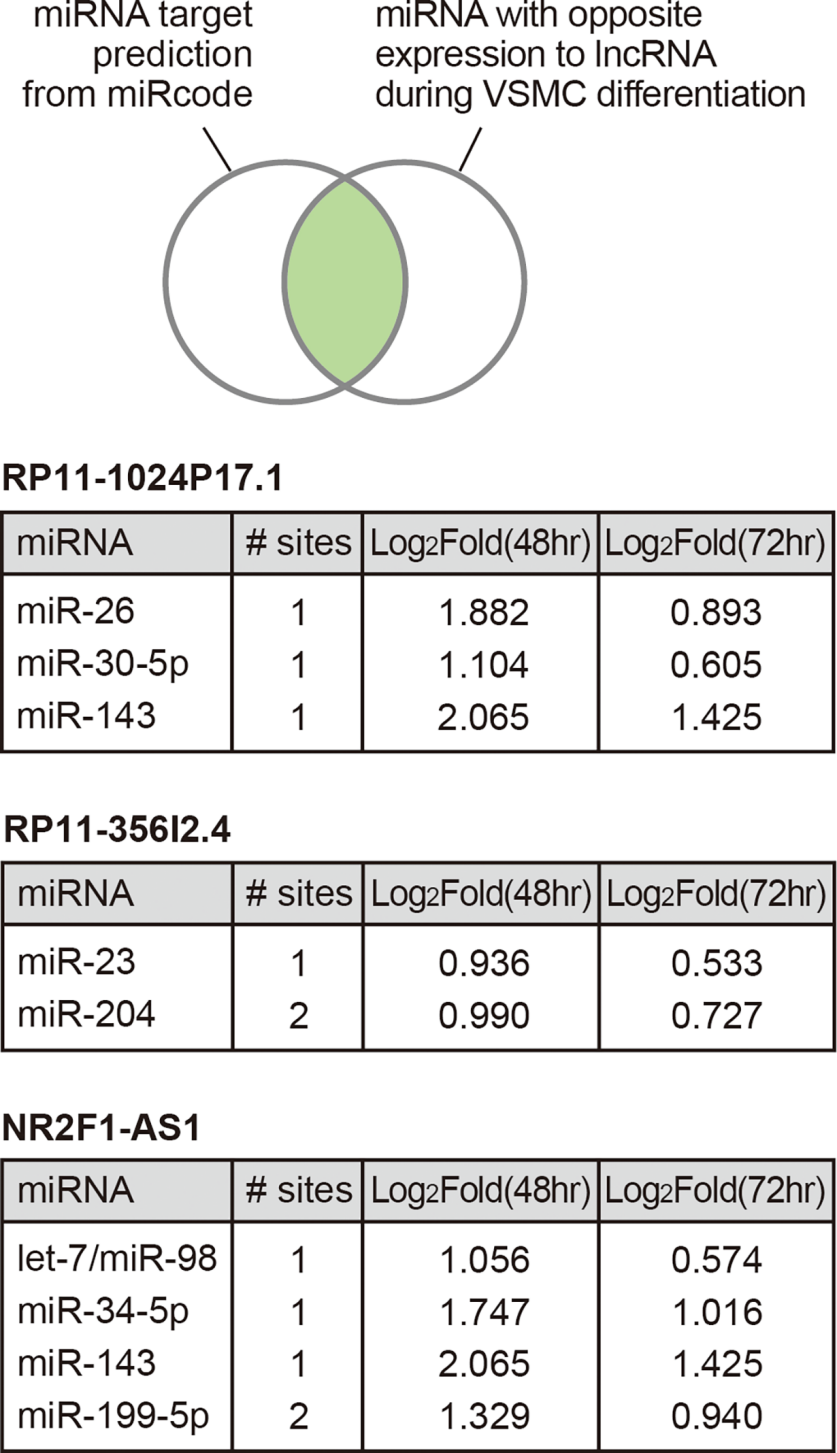
Analysis of lncRNAs as miRNA regulators. We intersected the list of lncRNA-targeting miRNAs predicted from miRcode with the list of miRNAs that shows opposite expression patterns to lncRNAs during VSMC differentiation, as depicted in the diagram (see Methods). For each lncRNA, selected miRNAs with binding sites at corresponding lncRNA and with inverse correlation in expression to the same lncRNA are shown. The number of miRNA-binding sites in each lncRNA sequence is indicated. The expression change of miRNAs between before and after the differentiation of VSMCs was re-calculated using the miRNA microarray data from GSE19544. The fold ratios from two different time points are indicated.

In addition to the role of lncRNAs as cis-acting transcriptional regulators, lncRNAs can work as competing endogenous RNAs by binding miRNAs in a sequence-specific manner and suppressing their function [9]. In this way, lncRNAs can increase the amount of target mRNAs that are originally suppressed by miRNAs. To identify miRNAs that could be regulated by selected lncRNAs above (Fig 4), we used miRcode, the database of miRNA target prediction [20]. To increase the reliability of the prediction, we utilized the expression data of miRNAs during VSMCs differentiation, which was obtained from GEO database (GSE19544) [21]. After obtaining the list of miRNAs that have binding sites in the lncRNA sequences, we only selected the miRNAs increased in differentiated phenotype because those three lncRNAs that we selected are decreased upon differentiation of VSMCs (Fig 4). We found several miRNAs for each lncRNA, which meet these criteria (Fig 6). Thus, these lncRNAs may regulate the function of miRNAs during phenotypic change of VSMCs by working as competing endogenous RNAs. All the miRNAs in this list were shown to be involved in the differentiation or other related phenotypes of smooth muscle cells from previous studies ([21, 34–40], suggesting the reliability of our analysis.

Finally, we searched NPInter database where the experimentally verified association between lncRNAs and proteins are documented [41]. Several interactions between lncRNAs selected above and RNA-binding proteins were found as shown in S4 Table. Among these RNA-binding proteins, ADAR1, hnRNPA1, and hnRNPA2/B1 were reported to be involved in the differentiation of smooth muscle cells [42–44]. Thus, our analyses suggest that the lncRNAs identified in this study indeed are expected to play important roles in the differentiation of VSMCs possibly through association with their binding proteins. Further researches are required to elucidate the regulatory mechanisms of these lncRNAs.

Although many lncRNAs have been discovered in diverse species and experimental models, their roles and pathological effects in muscles have not been studied as extensively as those related to other diseases, such as cancer. One of the issues in discovering lncRNAs or other types of noncoding RNAs in an experimental model is their inconsistency when analyzed using different approaches. Thus, lncRNAs identified in one approach may not be replicated in another approach, although both approaches are aimed at the same experimental model. The comparison of the expression profile of lncRNAs in the differentiation model of VSMCs confirmed this issue (Fig 1C). Although all the different treatments, including PDGF, MYOCD, and TGFβ, mimic the differentiation conditions of VSMCs, most lncRNAs showed inconsistent changes among these conditions. In addition, two of five lncRNAs that were consistently changed from these conditions showed different expression pattern in our serum withdrawal condition. In our previous study to identify miRNAs important in the progression of Alzheimer’s disease, we improved this problem by integrating various RNA-seq data sets from human patients, a mouse model, and a cellular model, and identified commonly changed miRNAs in these models [45]. In the present study, we applied a similar approach to identify lncRNAs that may have important roles in VSMCs differentiation by integrating the RNA-seq data from various differentiation conditions for VSMCs and confirming their expression change in yet another condition. From this analysis, we discovered dozens of lncRNAs with possible involvement in the phenotypic change of VSMCs, most of which have no previous reports of a link to the physiology of VSMCs (Fig 2). Moreover, for three lncRNAs, we confirmed the expression change in our differentiation system and analyzed their possible regulatory mechanism. Therefore, the list of lncRNAs identified in this study will be a useful resource for future research into the gene regulatory network during muscle differentiation.

## Supporting information

S1 Fig Heat map of long noncoding RNAs (lncRNAs) whose expression was increased in the contractile phenotype. The cluster indicated in Fig 1 with yellow box is magnified.
(PDF)

S2 Fig Heat map of long noncoding RNAs (lncRNAs) whose expression is increased in the synthetic phenotype. The cluster indicated in Fig 1 by a blue box is magnified.
(PDF)

S1 Table Long noncoding RNAs (lncRNAs) with differential expression during phenotypic change of vascular smooth muscle cells (VSMCs). For each lncRNA, their genomic locus (hg19), the fragments per kilobase of exon model per million mapped fragments (FPKM) values in each sample, log_2_ (fold change), and the *p* values from two-sided T test for each dataset, are shown. For those lncRNAs whose promoters were analyzed, the position of the transcription start sites and the strand of transcription are included. For the lncRNAs with incomplete annotation (red letters), transcription start sites were identified by manual curation using the information from RefSeq annotation. Only the significant lncRNAs from Fig 2 were included.
(XLSX)

S2 Table Long noncoding RNAs (lncRNAs) with chromatin immunoprecipitation sequencing (ChIP-seq) signals of transcription factors in their promoter regions. Among the transcription factors included in the ChIP-seq data from ENCODE, we identified those that were significantly enriched in the promoters of lncRNAs that increased in the synthetic phenotype compared with those increased in the contractile phenotype (one-sided Fisher’s exact test). This analysis resulted in three transcription factors with *p* values lower than 0.1. There was no transcription factor enriched in the lncRNA promoters of the contractile phenotype against those of the synthetic phenotype.
(XLSX)

S3 Table Bioinformatics analysis of coding potential of selected lncRNAs. The coding potential of selected lncRNAs in Figs 5 and 6 was analyzed at (A) CPAT and (B) CPC web servers [30, 31].
(XLSX)

S4 Table Interaction of selected lncRNAs with proteins. We used NPInter database to search the experimentally verified association between lncRNAs and proteins [41], and listed the pairs of lncRNA and interacting protein. The experimental method to identify these associations was also shown.
(XLSX)

S5 Table Primer sequences used in this study. (XLSX)
