# Supplementary figures and images for: Identification of long noncoding RNAs involved in muscle differentiation
Source: PLoS One. 2018 Mar 2;13(3):e0193898. doi: 10.1371/journal.pone.0193898 (PMC5834194; doi:10.1371/journal.pone.0193898)

Supplementary figure 1

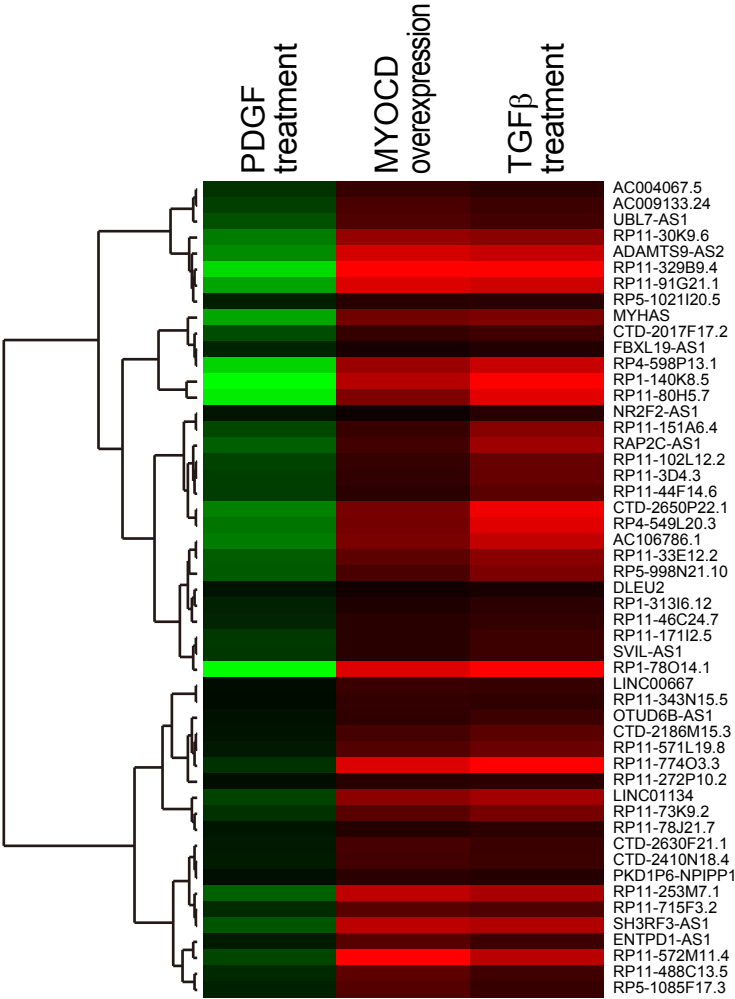

Supplement: S1 Fig — The cluster indicated in Fig 1 with yellow box is magnified. (PDF) [file pone.0193898.s001.pdf]

Supplementary figure 2

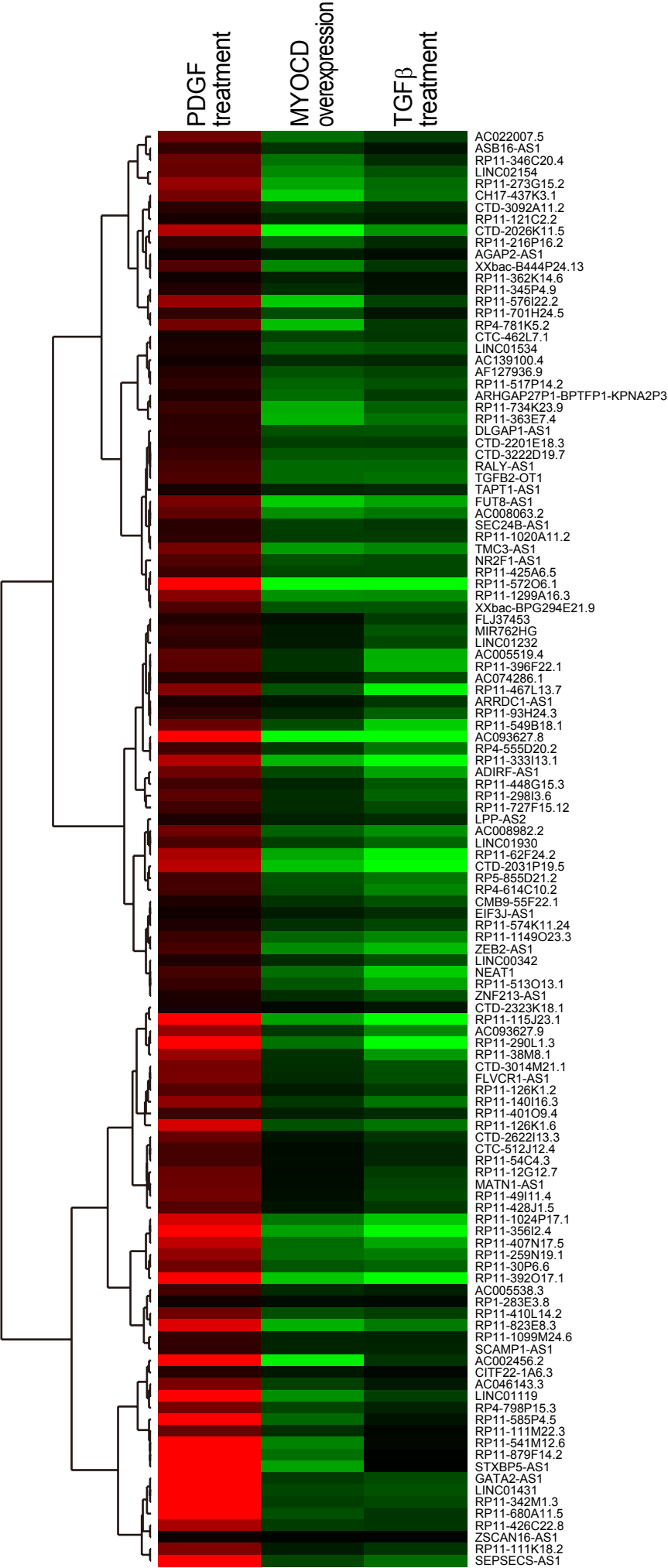

Supplement: S2 Fig — The cluster indicated in Fig 1 by a blue box is magnified. (PDF) [file pone.0193898.s002.pdf]
